# Resveratrol Improves Growth Performance, Intestinal Morphology, and Microbiota Composition and Metabolism in Mice

**DOI:** 10.3389/fmicb.2021.726878

**Published:** 2021-09-03

**Authors:** Yu Zhuang, Huijun Huang, Shuang Liu, Feng Liu, Qiang Tu, Yulong Yin, Shanping He

**Affiliations:** ^1^State Key Laboratory of Developmental Biology of Freshwater Fish, Hunan Provincial Key Laboratory of Animal Intestinal Function and Regulation, Hunan Normal University, Changsha, China; ^2^Jiangxi Provincial Key Laboratory for Animal Health, Institute of Animal Population Health, College of Animal Science and Technology, Jiangxi Agricultural University, Nanchang, China; ^3^Yucheng Baolikang Biological Feed Co., Ltd., Dezhou, China

**Keywords:** resveratrol, growth performance, intestinal morphology, microbiota community, weaning stress

## Abstract

**Background:**

Resveratrol (RSV) plays a vital role in alleviating various stresses and improving intestinal health. The current study was conducted to explore whether RSV alleviates weaning stress through improving gut health in a weaning mouse model. Forty 21-day-old weaned mice were randomly assigned to a control group without RSV treatment and three treatment groups with 10, 20, and 50 mg/kg RSV for 28 days.

**Results:**

The results showed that RSV at a dose of 20 mg/kg improved total body weight, intestinal morphology (villus length and the ratio of villus length to crypt depth), and the levels of intestinal barrier proteins (claudin-1 and occludin), but had little effect on the food intake, crypt depth, and serum free amino acids of mice. Compared with the control group, mice supplemented with RSV had decreased mRNA expression of genes related to inflammatory cytokines (IL-6 and IL-1β), but increased mRNA expression of genes related to host defense peptides (Defa3, Defa5, Defa20, and Lyz) and short-chain fatty acids (SCFAs) production (propionic acid, isobutyric acid, butyric acid, and isovaleric acid). In addition, 16S rRNA sequencing results showed that RSV supplementation increased the richness indices of intestinal microbiota (Chao, ACE) and shaped the composition of intestinal microbiota (e.g., increased β-diversity of intestinal microbiota community). Meanwhile, RSV supplementation increased genes of *Butyricicoccus*, *Ruminococcus_1*, and *Roseburia*, which are producers of SCFAs. Furthermore, RSV supplementation significantly influenced the metabolism of intestinal microbiota, namely, amino acids metabolism, lipid metabolism, and defense mechanisms.

**Conclusion:**

RSV can improve growth performance and intestinal morphology in weaning mice, possibly through improving gut immune response and microbiota function.

## Introduction

Weaning is a critical period for newborn mammals and profoundly affects gut health and the immune system (Melo et al., 2016; Furbeyre et al., 2017). During this phase, newborns are exposed to various stresses, such as lactational immunity depletion, immature gut, and changes in gut microbiota. As a consequence, weaning results in decreased feed intake, serious diarrhea, and depressed immune systems. Additionally, a number of studies have revealed that juvenile stress, such as weaning stress, has a profound impact on social behavior and metabolism in adulthood (Shtoots et al., 2018; Heard-Garris et al., 2019). For example, early life adversity (weaning stress) in piglets induces chronic functional diarrhea, intestinal permeability, and lasting alterations in gastrointestinal (GI) function (Pohl et al., 2017).

Recently, numerous studies have suggested that the gut ecosystem is involved in the pathogenesis of multiple diseases (Zhang et al., 2019). A growing number of evidence has suggested that gut microbiota is closely associated with host health (Patil et al., 2019; Ross et al., 2019). Gut microbiota provides enzymes to digest nutrients and interacts with host signaling pathways, thus affecting host metabolism and immunity through itself or microbial-derived metabolites. Furthermore, gut microbiota is tightly associated with the incidence of many chronic diseases (Cruz-Lebron et al., 2019; Lv et al., 2019), namely, diabetes, metabolic syndrome, and colon cancer. However, during the period of weaning, the state of the gut ecosystem is weakened and highly malleable. Early life establishment of infant gut microbiota sets the stage for adult microbiome and has profound effects on host metabolism and health. Therefore, improving infant gut ecology to profoundly affect the metabolism in adulthood has been the focus of recent studies.

Resveratrol (RSV) is a plant-derived stilbene that has various biological activities (Rauf et al., 2017; Zhang et al., 2018; Pan et al., 2019). Numerous studies have suggested that RSV acts on multiple cellular targets, namely, Nrf2, Sirt1, and AMPK, to control cellular processes and signaling pathways (Yang et al., 2018, 2019; Jiang et al., 2019). However, RSV has a high biotransformation rate in intestinal ecology, and a low dose of RSV is found in target tissues (Andres-Lacueva et al., 2012). A growing number of evidence has revealed that the health benefits of RSV could be associated with its capacity to alter the composition of the microbiota population, which was shown to have a profound impact on the health of organisms. For instance, supplementation of RSV could modulate gut microbiota, nutrient-sensing pathways, and oxidative stress to prevent the development of hypertension induced by maternal plus post-weaning high-fructose consumption (Tain et al., 2018). Therefore, RSV can act on gut microbiota to affect body metabolism.

Few studies have investigated the effects of dietary RSV on both the complex gut microbiota in mice programmed by post-weaning and the metabolism during juvenile phase. Therefore, the present study was conducted to explore the effects of RSV on growth performance, intestinal morphology, intestinal barrier functions, serum amino acid profiles, short-chain fatty acids (SCFAs), and microbiota community of C57BL/6 mice exposed to post-weaning juvenile stress. Our results will provide new insight into the mechanisms by which RSV regulates intestinal microecology in juvenile mice.

## Materials and Methods

### Animals

All the procedures involving mice in this study were approved by the Animal Care and Use Committee of Hunan Normal University, Changsha City, Hunan, China. Three-week-old C57BL/6 mice (without received diet) were obtained from Hunan SLAC Laboratory Animal Co., Ltd. (Changsha, China) and were kept in an environmentally controlled room (temperature: 24 ± 2°C, humidity: 60 ± 5%) with a 12-h light–dark cycle. For all the experimental period, all the mice were housed in individual cages and had *ad libitum* access to food and water.

### Experimental Design and Treatments

Three-week-old C57BL/6 mice (11 ± 1 g) were randomly assigned into four groups (*n* = 10/group): 1) control group (basal diet + vehicle solution), 2) RSV10 (basal diet + 10 mg/kg/day RSV), 3) RSV20 (basal diet + 20 mg/kg/day RSV), and 4) RSV50 (basal diet + 50 mg/kg/day RSV). The experiment was conducted for 4 weeks (28 days), and all the treatments were performed by gavage during the experimental period (28 days). The treatments of RSV10, RSV20, and RSV50 received RSV (Selleckchem, Houston, TX, United States) at a dose of 10, 20, and 50 mg/kg, respectively. Due to its low solubility in water, RSV was suspended into 0.5% (w/v) carboxymethylcellulose solution (Sigma-Aldrich, St. Louis, MO, United States) according to a previous study (Yang et al., 2019). During the experimental period, mice of all the treatments were daily recorded to calculate average daily feed intake and total body weight gain.

### Sample Collection

After 4 weeks of RSV gavage daily, animals were then humanely sacrificed, and samples of blood were taken as in a previous report (Ren et al., 2018). For collection of the duodenum, jejunum, and ileum, the middle part of the duodenum, jejunum, and ileum samples was collected after washing with phosphate buffer saline (PBS, pH = 7.4). Samples of the ileum were collected for histological analysis, and cecum contents were collected for microbiota community analysis. Additionally, before sacrifice, the fresh feces from each group were collected. All methods and practical steps were in line with animal welfare rules in China.

### Tissue Histological Examination

Ileums were stained with hematoxylin and eosin (H&E staining). Briefly, the cross-sections of ileum samples were preserved in 4% formaldehyde and then dehydrated and embedded using standard paraffin embedding techniques. Section of 5 μM was cut and stained with H&E staining. The villus height and crypt were measured under a microscope × 40 combined magnification using Image-Pro Plus for image processing and analysis system.

### Determination of Serum Amino Acid Profiles

Free amino acid profiles in serum were determined according to our previous study (Ren et al., 2018). Briefly, the same volume of serum and 2.5% sulfosalicylic acid were added to a sealed centrifuge container. The two substances were shaken together and allowed to stand overnight at 4°C, and the containers were centrifuged at 1,000 rpm for 15 min at 4°C. Following this process, the supernatant was filtered through a 0.22-μM membrane and analyzed using Hitachi L-8900 automatic amino acid analyzer according to the manufacturer’s instructions.

### Determination of SCFAs

Fresh fecal samples were collected for determination of SCFAs (acetic acid, propionic acid, butyrate, isobutyric acid, valerate, and isovaleric acid) by using the Agilent 6890 gas chromatography (Agilent Technologies, Inc., Palo Alto, CA, United States) according to our previous study (Ren et al., 2018).

### Quantitative Real-Time PCR

Total RNA of ileum samples from mice was extracted using TRIzol reagent (TaKaRa, Dalian, China), and first-stand cDNA was synthesized using a reverse transcription kit (TaKaRa, Dalian, China) following the manufacturer’s instruction. Quantitative real-time PCR (qRT-PCR) was performed on QuantStudio^TM^ 5 Real-Time PCR System analyzer (Applied Biosystems, Foster City, CA, United States) to quantify mRNA expression. The details of gene in this study are summarized in Table 1 and synthesized by TsingKe Biological Technology (Changsha, China). The expression of β-actin was analyzed as an internal control for the normalization of the results. Relative expression levels were calculated using the 2^–ΔΔCt^ method according to a previous study (Yin et al., 2018).

**TABLE 1:** Sequences of target genes’ primers.

Gene names	Genes No.	Sequence of primer	Length (bp)
Claudin-1	NM_016674.4	ACTGTGGATGTCCTGCGTTT	258
		ACTAATGTCGCCAGACCTGAA	
Occludin	NM_001360536.1	CAGGTGAATGGGTCACCGAG	163
		CCAAGATAAGCGAACCTGCC	
ZO-1	NM_009386.2	CTTCCCGGACTTTTGTCCCA	220
		CATTGCTGTGCTCTTAGCGG	
IL-6	NM_031168.2	CTCTGCAAGAGACTTCCATCCA	124
		GACAGGTCTGTTGGGAGTGG	
IL-1β	NM_008361.4	TGCCACCTTTTGACAGTGATG	220
		AAGGTCCACGGGAAAGACAC	
TNF-α	NM_001278601.1	AGGCACTCCCCCAAAAGATG	213
		CCACTTGGTGGTTTGTGAGTG	
MUC2	NM_023566.3	CCTGAAGACTGTCGTGCTGT	100
		GGGTAGGGTCACCTCCATCT	
MUC3	NM_010843.1	AGTGCTGTTGGTGATCCTCG	193
		AGAGTCCAGGGGCATGTAGT	
Retnlβ	NM_023881.4	TGGCTGTGGATCGTGGGATA	96
		TAAACCATTCGGCAGCAGCG	
Lyz	NM_013590.4	GAGACCGAAGCACCGACTATG	214
		CGGTTTTGACATTGTGTTCGC	
ang4	NM_177544.4	TGGCCAGCTTTGGAATCACTG	151
		GCTTGGCATCATAGTGCTGACG	
Defa3	NM_007850.2	TCCTCCTCTCTGCCCTCGT	240
		GACCCTTTCTGCAGGTCCC	
Defa5	NM_007851.2	GTCCAGGCTGATCCTATCCA	202
		GATTTCTGCAGGTCCAAAA	
Defa20	NM_183268.4	GACCTGCTCAGGACGACTTT	94
		GCCTCAGAGCTGATGGTTGT	
meprinβ	NM_008586.2	CAGGCAAGGAACACAACTTC	118
		TCTGTCCCGTTCTGGAAAG	
Mmp7	NM_010810.5	CTGCCACTGTCCCAGGAAG	175
		GGGAGAGTTTTCCAGTCATCG	
CFTR	NM_021050.2	AAGGCGGCCTATATGAGGTT	107
		AGGACGATTCCGTTGATGAC	
NKCC	NM_009194.3	CAAGGGTTTCTTTGGCTAT	144
		TCACCTGAGATATTTGCTCC	
SLC26A	NM_021353.3	TTCCCCTCAACATCACCATCC	111
		GTAAAATCGTTCTGAGGCCCC	
NHE3	NM_001033289.2	TGGCAGAGACTGGGATGATAA	145
		CGCTGACGGATTTGATAGAGA	
Ano1	NM_178642.5	AGCAGGCTTCTGACCATCAC	99
		CACGTCCAGACGACACAAGA	
PPARr	NM_001127330.2	CCAGCATTTCTGCTCCACAC	93
		ATTCTTGGAGCTTCAGGCCA	
GAPDH	XM_017321385.1	CTTATCAGGCCAAGTATGATG	96
		CAACCTGGTCCTCAGTGTAGC	

### Western Blot

Total cellular protein of IPEC-J2 cells was extracted by RIPA (Beyotime, Shanghai, China) with protease inhibitor and phosphatase inhibitor cocktail (Selleckchem, Houston, TX, United States). The content of protein was quantified using BCA protein assay kit (Beyotime, Shanghai, China) according to the instructions. And then the extracts of tissues were protein denaturation incubation with SDS-loading buffer at 100°C for 15 min. The denaturation protein was resolved over SDS-PAGE, transferred into 0.45 μM PVDF membranes, and then blocked with 5% non-fat dry milk for 2 h at room temperature, followed by incubation with primary antibodies for detection of claudin-1, zonula occludens-1 (ZO-1), and occludin-1. The membranes were probed by the secondary antibodies for 1.5 h at room temperature. And the blots were visualized by the enhancing chemiluminescence (ECL) method. The blots were quantified by measuring Image Lab^TM^ Software (Bio-Rad, Hercules, CA, United States).

### Microbial DNA Isolation and Microbiota Analysis Based on 16S RNA High-Throughput Sequencing

Total bacterial DNA of cecum content was extracted using a QIAamp DNA Stool Mini Kit (Qiagen, Hilden, Germany) according to the manufacturer’s instructions. The diversity and composition of the bacterial community were determined by high-throughput sequencing of the microbial 16S rRNA genes. The V3–V4 hypervariable region of the 16S rRNA genes was PCR amplified using primers, 338F: 5′-ACTCCTACGGGAGGCAGCA-3′ and 806R: 5′-GGACTACHVGGGTWTCTAAT-3′. Paired-end sequencing was performed on the Illumina HiSeq 2500 platform (BioMarker, Beijing, China). Raw 16S data sequences were obtained before being screened and assembled using the Trimmomatic (v 0.33) (Caporaso et al., 2010) and FLASH software packages. UCHIME (V4.2) was used to analyze the high-quality sequences and determine operational taxonomic units (OTUs). Subsequently, high-quality sequences were aligned against the SILVA reference database1 and clustered into OTUs at a 97% similarity level using the UCLUST algorithm2. Each OTU was assigned to a taxonomic level with the Ribosomal Database Project Classifier program v2.203.

### Statistical Analyses

Results from a representative of three independent experiments were expressed as means ± standard error of the mean (SEM). Data among all treatments were analyzed by one-way analysis of variance (ANOVA) followed by Dunnett’s multiple comparisons if the data were Gaussian distribution and had equal variance or analyzed by Kruskal–Wallis followed by Dunn’s multiple comparisons if the data were not normally distributed. The Gaussian distribution of data was analyzed by D’Agostino–Pearson omnibus normality test, and Kolmogorov–Smirnov test was tested for multiple comparisons; a value of *p* < 0.05 was accepted as statistically significant. The statistical analyses were performed by GraphPad Prism 7. In addition, the statistical analyses of the microbial community were performed using the R package software (version 2.15.3; R Core Team, Auckland, New Zealand), and the remaining data were performed using BMKCloud^1^.

## Results

### Growth Performance and Intestinal Morphology of Post-weaning Mice Fed With RSV

**FIGURE 1:**
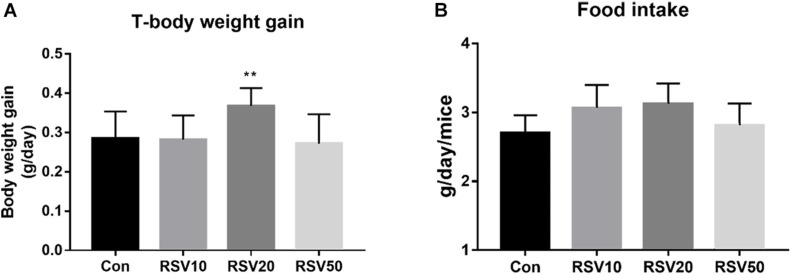
Effect of RSV on body weight and feed intakes of weaned mice. **(A)** Average daily gain per mice in each group. **(B)** Average daily feed intake of mice in each group. Con = control group, RSV10 = 10 mg/kg RSV, RSV20 = 20 mg/kg RSV, RSV50 = 50 mg/kg RSV. **Means with double asterisks are significantly different (*p* < 0.01) from values of control group, and all the data are expressed as the means ± SEM.

**FIGURE 2:**
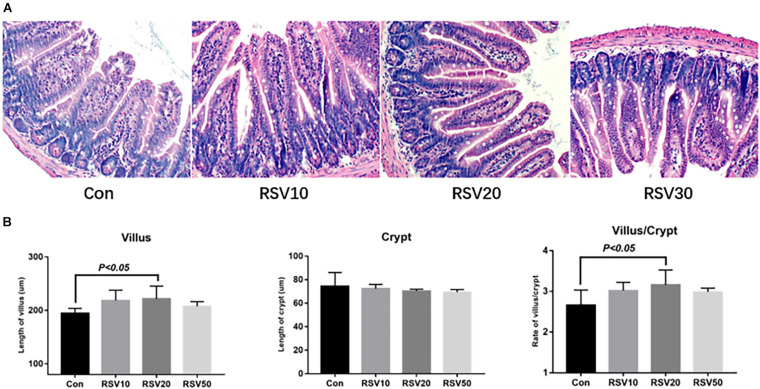
Effect of RSV on intestinal morphology of weaned mice. **(A)** Ileum morphology of mice in each group was evaluated by hematoxylin and eosin (HE) staining. **(B)** Villus length, crypt depth, and the ratio of villus length to crypt depth of RSV-treated mice. Con = control group, RSV10 = 10 mg/kg RSV, RSV20 = 20 mg/kg RSV, RSV50 = 50 mg/kg RSV. All the data are expressed as the means ± SEM.

Mice fed with 20 mg/kg RSV had increased (*p* < 0.05) body weight gain compared with mice in the control group during the experimental period, while there was no significant difference (*p* > 0.05) in food intake among each group (Figure 1). In addition, mice fed with 20 mg/kg RSV had increased (*p* < 0.05) ileal villus height and ratio of villus height to crypt depth compared with those mice in the control, 10 and 50 mg/kg RSV groups. However, no differences were observed in crypt depth (*p* > 0.05) among each group (Figure 2).

### Effects of Dietary RSV on Serum Amino Acids Profile, Intestinal Barrier Function, and Cytokine Expression in Post-weaning Mice

The contents of taurine (Tau), Gly, and Phe in the serum of mice fed with RSV were significantly increased (*p* < 0.05) compared with those in the control group. In contrast, the contents of Thr, Ser, Arg, and Glu in the serum of mice fed with RSV were significantly decreased (*p* < 0.05) compared with those in the control group. However, there were no significant differences (*p* > 0.05) in the contents of Ala, Val, Met, Leu, Ile, Lys, and Orn in the serum among each treatment (Table 2).

**TABLE 2:** Effect of resveratrol on the serum amino acid profiles in weaned mice.

Items (mM)	CON^a^	RSV10^b^	RSV20^c^	RSV50^d^	*P*-value
Tau	0.4791	0.5928*	0.6256*	0.5331*	<0.001
Urea	5.164	5.985*	5.245	4.989	0.0117
Asp	0.0128	0.011	0.009143*	0.0115	0.0642
Thr	0.112	0.081*	0.07686*	0.0745*	0.0001
Ser	0.07667	0.0619*	0.05429*	0.0627*	0.0029
Glu	0.191*	0.1202	0.1848	0.1369*	0.0379
Gly	0.0738	0.0924*	0.08686	0.0997*	0.0130
Ala	0.211	0.1684	0.1724	0.1799	0.1152
Cit	0.028	0.036*	0.0332	0.02956	0.0172
Val	0.1133	0.1187	0.108	0.1213	0.5935
Met	0.047	0.0295	0.02457	0.0258	<0.0001
lle	0.04	0.0444	0.04114	0.04943	0.5872
Leu	0.05267	0.0663	0.05629	0.07983*	0.0052
Tyr	0.0365	0.02967	0.03214	0.02538*	0.0008
Phe	0.05233	0.0629	0.1132*	0.0645*	<0.0001
Orn	0.051	0.0517	0.05314	0.053	0.9137
Lys	0.2603	0.1797	0.162	0.171	<0.0001
Arg	0.09533	0.0763*	0.06725*	0.0735*	<0.0001

*^*a*^Con = control group.*

*^*b*^RSV10 = 10 mg/kg RSV.*

*^*c*^RSV20 = 20 mg/kg RSV.*

^*d*^RSV50 = 50 mg/kg RSV.

*Means with single asterisks are significantly different (*p* < 0.05) from values of control group, and all the data are expressed as the means of six samples.

**FIGURE 3:**
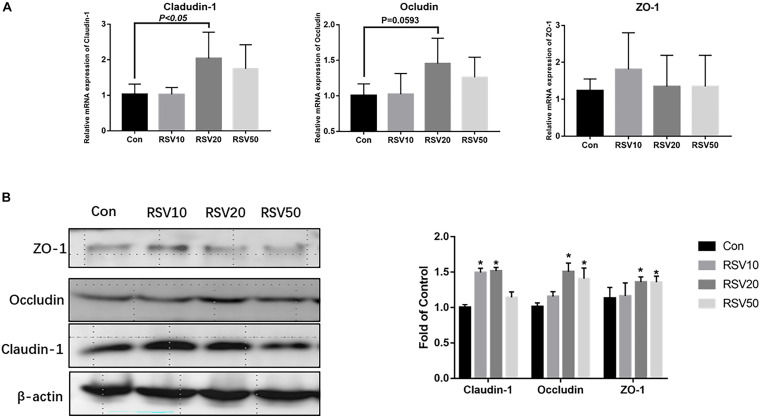
Effect of RSV on the levels of intestinal barrier proteins in weaned mice. **(A)** The relative mRNA expression of claudin-1, occludin, and ZO-1 was detected by qRT-PCR. **(B)** The protein levels of Claudin-1, Occludin and ZO-1 were detected by Western blot with β-actin as the loading control. Con = control group, RSV10 = 10 mg/kg RSV, RSV20 = 20 mg/kg RSV, RSV50 = 50 mg/kg RSV. *Means with single asterisks are significantly different (*p* < 0.05) from values of control group, and all the data are expressed as the means ± SEM.

**FIGURE 4:**
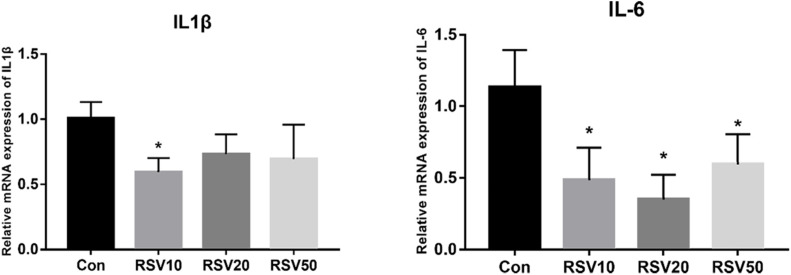
Effect of RSV on the expression of IL-1β and IL-6 in weaned mice. The relative mRNA expression of IL1β and IL-6 was detected by qRT-PCR. Con = control group, RSV10 = 10 mg/kg RSV, RSV20 = 20 mg/kg RSV, RSV50 = 50 mg/kg RSV. *Means with single asterisks are significantly different (*p* < 0.05) from values of control group, and all the data are expressed as the means ± SEM.

qRT-PCR and Western blot analysis showed that the expression of claudin-1 and occludin in mice fed with 20 mg/kg was significantly (*p* < 0.05) increased in the ileum compared with that in the control group (Figures 3A,B). In contrast, there was no difference (*p* > 0.05) in the expression of ZO-1 in the ileum among each treatment (Figures 3A,B). In addition, the mRNA expression of IL-6 and IL-1β in the ileum of mice fed with RSV was significantly decreased compared with that in the control group (Figure 4).

### Effect of Dietary RSV on the Expression of Intestinal Ionic Transporter-Related Genes and Antimicrobial Peptide Genes

There were no differences (*p* > 0.05) in the mRNA expression of NKCC, SLC26A2, ANO1, and NHC3 in the ileum among each treatment (Supplementary Figure 1), while the mRNA expression of CFTR was significantly decreased (*p* < 0.05) in mice fed with RSV compared with that in the control group (Supplementary Figure 1).

**FIGURE 5:**
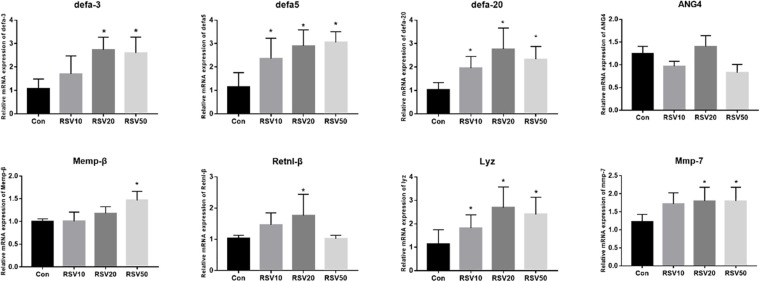
Effect of RSV on the expression of AMP genes in the ileum of weaned mice. The relative mRNA expression of AMP related genes was detected by qRT-PCR. Con = control group, RSV10 = 10 mg/kg RSV, RSV20 = 20 mg/kg RSV, RSV50 = 50 mg/kg RSV. *Means with single asterisks are significantly different (*p* < 0.05) from values of control group, and all the data are expressed as the means ± SEM.

The mRNA expression of Lyz, mmp7, Defa3, Defa5, and Defa20 in the ileum of mice fed with 20 and 50 mg/kg RSV was significantly (*p* < 0.05) increased compared with that in the control group. In addition, compared with mice in the control group, the mRNA expression of Memp-β and Retnl-β was significantly increased (*p* < 0.05) in the ileum of mice fed with 50 and 20 mg/kg RSV, respectively (Figure 5).

### Effect of Dietary RSV on the Production of Fecal SCFAs in Post-weaning Mice

**FIGURE 6:**
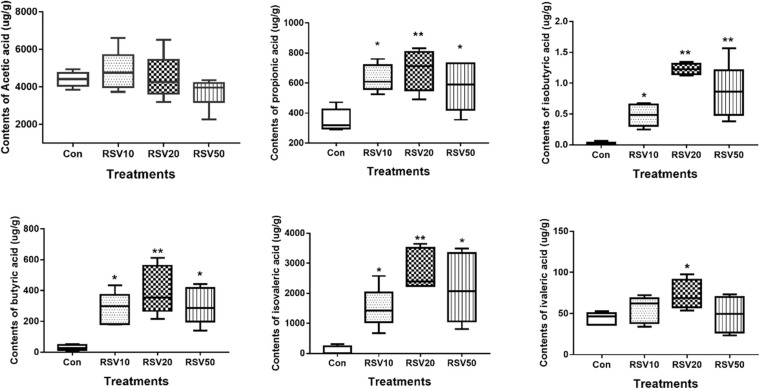
Effect of RSV on the production of fecal SCFAs in weaned mice. The production of fecal SCFAs was determined using the Agilent 6890 gas chromatography. Con = control group, RSV10 = 10 mg/kg RSV, RSV20 = 20 mg/kg RSV, RSV50 = 50 mg/kg RSV. *Means with single asterisks are significantly different (*p* < 0.05) from values of control group, and **means with double asterisks are extremely significantly different (*p* < 0.01) from values of control group. All the data are expressed as the means ± SEM.

The contents of propionic acid, isobutyric acid, butyric acid, and isovaleric acid in the feces of mice fed with RSV were significantly increased (*p* < 0.05) compared with those in the control group. The contents of valeric acid in mice fed with 20 mg/kg RSV were significantly increased (*p* < 0.05) compared with those in the control group, whereas there were no differences (*p* > 0.05) in the contents of acetic acid in the feces among each treatment (Figure 6).

### Microbial Assessment and Metabolic Function Prediction in the Cecum Content of Post-weaning Mice Fed With RSV

**FIGURE 7:**
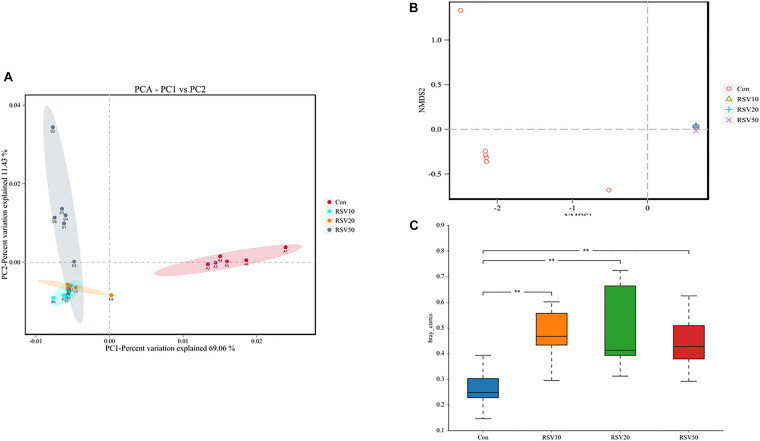
Effect of RSV on Beta diversity indices of bacterial communities in the cecum content of weaned mice. **(A)** The principal component analysis (PCA) and **(B)** nonmetric multidimensional scaling (NMDS) plots were used to analyze bacterial communities. **(C)** Weighted Unifrac analysis was used to measure β-diversity. Con = control group, RSV10 = 10 mg/kg RSV, RSV20 = 20 mg/kg RSV, RSV50 = 50 mg/kg RSV. **Means with double asterisks are extremely significant different (*p* < 0.01) from values of control group.

**FIGURE 8:**
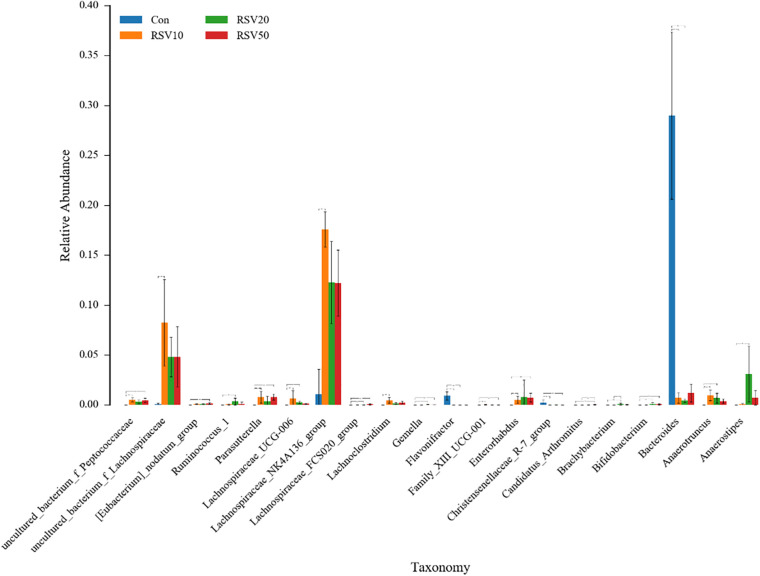
Effect of RSV on the bacterial communities in the cecum content of weaned mice at the genus level. Con = control group, RSV10 = 10 mg/kg RSV, RSV20 = 20 mg/kg RSV, RSV50 = 50 mg/kg RSV. *Means with single asterisks are significantly different (*p* < 0.05) from values of control group, and **means with double asterisks are extremely significant different (*p* < 0.01) from values of control group.

Rarefaction curves of all the samples showed that the selected sequences were sufficient to determine the majority of bacterial diversity parameters (Table 3). The indices of Chao, ACE, and Simpson in mice fed with RSV were significantly increased compared with the control group, while the indices of Shannon in mice fed with RSV were significantly decreased compared with the control group. Meanwhile, the relative abundance of *Firmicutes*, *Proteobacteria*, and *Actinobacteria* was significantly increased in mice fed with RSV compared with the control group, whereas other phyla, namely, *Bacteroidetes* and *Verrucomicrobia*, were significantly decreased in mice fed with RSV compared with the control group (Supplementary Figure 2). Principal component analysis (PCA), non-metric multidimensional scaling (NMDS) plots, and the two-tailed Student’s *t*-test statistical analysis of β-diversity weighted Unifrac revealed obvious segregation between the control and RSV-treated groups (Figure 7). The relative abundance of microbial community genus analyzed by rank-sum test was shown in Figure 8. *Lactobacillus* (probiotics of genus), *Anaerotruncus*, and *Ruminococcus_1* (SCFA producers of genus) were significantly increased in mice fed with 20 mg/kg RSV compared with the control group. In addition, the fermentation genus of the SCFAs of *Butyricicoccus*, *Roseburia*, *Bifidobacterium*, and *Anaerotruncus* (beneficial genus) was significantly increased, whereas the *Streptococcus* (the harmful bacteria genus) in mice fed with RSV was significantly decreased compared with the control group (Supplementary Table 1).

**TABLE 3:** Alpha diversity indices of bacterial communities in the cecum content of weaned mice.

Item	Experimental groups				*P*-value
	
	Con^a^	RSV10^b^	RSV20^c^	RSV50^d^	
Coverage	>99.9%	>99.9%	>99.9%	>99.9%	
Chao	142.8	331.8	331.8	316.9	<0.0001
ACE	170.9	323.3	328.4	315	<0.0001
Shannon	0.1956	0.4827	0.03617	0.06407	<0.0001
Simpson	2.152	4.089	4.169	3.867	<0.0001

*^*a*^Con = control group.*

*^*b*^RSV10 = 10 mg/kg RSV.*

*^*c*^RSV20 = 20 mg/kg RSV.*

*^*d*^RSV50 = 50 mg/kg RSV.*

*All the data are expressed as the means of six samples.*

**FIGURE 9:**
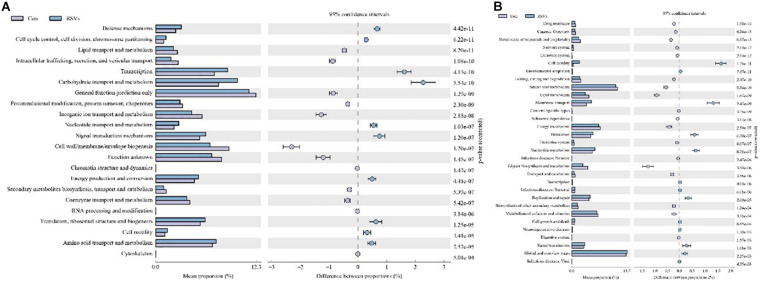
Effect of RSV on predictive functional profiling of microbial communities in the cecum content of weaned mice at the genus level. **(A)** The KEEG genus level and **(B)** COG genus level were generated by OTU annotation by PICRUSt software analysis. Con = control group, RSVs = 10 mg/kg RSV, RSV20 = 20 mg/kg RSV and RSV50 = 50 mg/kg RSV group.

To investigate further changes in microbial metabolic functions in the cecum microbiota induced by RSV treatment, we used PICRUSt to generate the metagenome based on 16S RNA sequencing results. We found that 31 metabolism pathways showed significant differences among the groups at KEGG level 3 (genus level), including those associated with amino acid metabolism, lipid metabolism, membrane transport, energy metabolism, cell growth and death, and metabolism of cofactors and vitamins (Figure 9A). Furthermore, 31 function enrichments showed significant differences among the groups at KEGG level 3, including those associated with defense mechanisms, lipid transport and metabolism, carbohydrate transport and metabolism, nucleotide transport and metabolism, and amino acid transport and metabolism (Figure 9B).

## Discussion

Weaning is a critical period for newborn mammals, and this period deeply affects gut health and the immune system. During this phase, gut ecology tends to be mature and has a profound effect on host metabolism. Recent studies have shown that supplementation of RSV can modulate gut microbiota, nutrient-sensing pathways, and oxidative stress to prevent the development of hypertension programmed by maternal plus post-weaning high-fructose consumption in early life of newborn (Hsu et al., 2019; Zou et al., 2019). In the present study, our results showed that supplementation of RSV in weaned mice had better growth performance, higher SCFAs in feces, and increased expression of AMP genes.

The intestine is the first defense barrier against various stresses and also a selective barrier that absorbs small peptides, amino acids, vitamins, and so on. Given the improvement of growth performance in mice fed with RSV, it is reasonable to expect that the intestinal morphology of mice may be altered. The intestinal villus height and VH:CD ratio are widely used as important indicators to assess the capacity of nutrient digestion and absorption in the small intestine (Zou et al., 2019). The present study showed that supplementation of 20 mg/kg RSV had higher villus height and VH:CD ratio in the ileum than the control group, which is consistent with body weight increase in those groups. In addition, RSV could reduce oxidative stress-induced apoptosis of intestinal epithelial cells (Storniolo and Moreno, 2019); thus, the results of villus height and VH:CD ratio in mice fed with RSV are consistent with our expectation. Furthermore, Tau, as sulfamic acid, does not participate in protein synthesis and is widely considered to have antioxidant activity (Yamashita et al., 2019). In the present study, the contents of Tau were significantly increased in the serum of mice fed with RSV compared with those in the control group. Meanwhile, the contents of Gly and Phe in mice fed with RSV were also elevated. A previous study has revealed that Gly and Phe not only are building blocks of proteins but also have many physiological effects, including acting as antioxidants and regulation of hormone secretion and metabolism (Valdes et al., 2019). It should be noted that RSV can reduce the contents of Arg and Glu in serum possibly through affecting intestinal amino acid transporter and competing the absorption of amino acids.

Barrier is a fundamental function of intestinal epithelium to limit interactions between luminal contents, such as microbiota and antigens, and the host. Tight junctions are the principal determinants of barrier function in the intact epithelia and are composed of transmembrane and cytosolic proteins. Claudin-1, occludin, and ZO-1 are major transmembrane and cytosolic proteins (Hsu et al., 2019). Our data showed that supplementation of RSV had higher expression of claudin-1, occludin, and ZO-1 in the ileum than the control group. Many studies have shown that RSV can maintain gut barrier integrity and upregulate the expression of tight junction proteins *in vivo* and *in vitro* (Wang et al., 2016; Zhang et al., 2017). However, Muc-2, the large molecular weight glycoprotein secreted by goblet cells, was not affected in our study. It suggests that RSV has no effect on intestinal chemical barrier, which is consistent with goblet cell results in the colon (data not shown).

Cytokines play important roles in the immune and inflammatory response. Previous studies have shown that dietary RSV has beneficial effects on regulating inflammatory response and innate immunity (Li et al., 2019; Meng et al., 2019; Yang et al., 2019). The present study showed that the expression of IL-1β and IL-6 was significantly inhibited by dietary RSV, which is consistent with previous studies on various pathological models *in vivo* and *in vitro* (Tain et al., 2018; Zhuang et al., 2019). In addition, the intestine functions as a main secretory organ, which maintains the homeostatic control of fluid and electrolytes balance and affects host defenses and microbiota in the gut. However, dietary RSV showed little effect on the expression of the genes regulating intestinal secretion, such as NKCC, SLC26A2, ANO1, and NHC3. The expression of CFTR, a cAMP-activated epithelial Cl^–^ and HCO_3_^–^ channel, was significantly decreased in mice fed with RSV. CFTR plays a pivotal role in intestinal secretion and regulation of bacterial colonization in the intestine. Moreover, the expression of major AMP genes, such as defensins and Lyz, was significantly elevated in mice fed with RSV compared with the control group. In addition, the expression of Mmp-7, which is responsible for the maturation of AMP genes (Tomas et al., 2016; Cazorla et al., 2018), was correlated with the upregulation of AMP-coding genes tested in the ileum, such as defensins and Lyz. It seemed that dietary RSV induced a global increase in mucosal defense. Meanwhile, previous studies have revealed that the expression of AMP genes is correlated with spatial distribution of bacteria. Hence, intestinal microbe and their metabolites in mice fed with RSV have been assayed in our study.

Recent studies have revealed that microbiome and their metabolites are increasingly recognized to potentially impact host physiology by participating in digestion and absorption of nutrients, shaping of the mucosal immune response, and producing or modulating a plethora of potentially bioactive compounds, such as SCFAs (Rooks and Garrett, 2016; Bain and Cerovic, 2020). In the present study, our results showed that the contents of SCFAs, such as propionic acid, isobutyric acid, butyric acid, and isovaleric acid, were significantly increased in mice fed with RSV. Correspondingly, the composition of microbiome in mice fed with RSV also changed. SCFAs are mainly produced in the hindgut of animals (colon and cecum) and are metabolic end products produced by anaerobic microorganisms fermenting indigestible compounds through the glycolysis pathway and the pentose phosphate pathway. Previous studies have revealed that SCFAs play vital roles in regulating cell proliferation and differentiation and maintaining intestinal mucosal integrity (Rooks and Garrett, 2016). Juanola et al. (2018) revealed that SCFAs can regulate intestinal immune function and promote T cell differentiation and immune factor secretion through its G-protein coupled receptor (GPCR). Supplementation of berberine or other plant extracts in mice results in an increment of SCFA contents (Wang et al., 2017), which is consistent with our results. In addition, *Butyricicoccus*, *Ruminococcus_1*, and *Roseburia*, which are recognized as SCFA producers (Rooks and Garrett, 2016; Lv et al., 2018), were significantly increased in mice fed with RSV.

Newborn gut microbiota has a simple and distinct microbial composition. Furthermore, immature gut microbiota is sensitive to environmental factors and vulnerable to be disturbed. Using 16S rDNA high-throughput sequencing, we found that the dominant phylum of cecal contents was *Bacteroidetes*, *Firmicutes*, *Verrucomicrobia*, *Proteobacteria*, and *Actinobacteria*, which is in line with previous studies. Microbial diversity, evidenced by Chao1, ACE, Shannnon, and Simpson indexes, showed a significant change in mice fed with RSV. While the β-diversity, evidenced by PCA and NMDS, also indicated that differences in overall diversity among individual mice became greater with dietary RSV. As previous studies reported, higher microbial diversity indicates a better intestinal condition and physiological prevention of exogenous bacterial colonization and environmental stresses (Chen et al., 2017; Zhou et al., 2019). Furthermore, *Bifidobacterium* and *Anaerotruncus* showed a significant increase, but *Streptococcus* showed a remarkable decrease in the RSV group compared with the control group. Therefore, supplementation of RSV in mice showed a better intestinal condition to prevent the colonization of harmful bacteria. The PICRUSt analysis by KEGG and COG predicted that RSV may alter nutrient metabolism, such as energy production, lipid transport, and amino acid transport through changing intestinal microbial function of weaning mice. Meanwhile, the host defense was also altered in function prediction analysis, such as defense mechanisms, infectious diseases: parasitic, and infectious diseases: bacterial. Therefore, the results of PICRUSt analysis were highly consistent with current results of growth performance, SCFAs, and host defenses.

## Conclusion

Collectively, our results indicate that mice supplemented with RSV show a greater growth performance and ileum morphology. In addition, supplementation of RSV induces the production of SCFA and the expression of AMP genes and decreases the expression of inflammatory genes. Meanwhile, supplementation of RSV also affects the bacterial community diversity and microbial function in mice cecum. This study will provide valuable guidance for the potential of using RSV as an additive against post-weaning juvenile stress.

## Data Availability Statement

The data presented in the study are deposited in the repository (https://www.ncbi.nlm.nih.gov/), accession number (PRJNA596657).

## Ethics Statement

All procedures involving mice in this study were approved by the Animal care and Use Committee of Hunan Normal University, Changsha city, Hunan, China.

## Author Contributions

YZ, YY, and SH contributed to conception and design of the study. HH organized the database. SL performed the statistical analysis. YZ wrote the first draft of the manuscript. FL and QT wrote sections of the manuscript. All authors contributed to manuscript revision, read, and approved the submitted version.

## Conflict of Interest

FL and QT are employed by Yucheng Baolikang Biological Feed Company Limited. The remaining authors declare that the research was conducted in the absence of any commercial or financial relationships that could be construed as a potential conflict of interest.

## Publisher’s Note

All claims expressed in this article are solely those of the authors and do not necessarily represent those of their affiliated organizations, or those of the publisher, the editors and the reviewers. Any product that may be evaluated in this article, or claim that may be made by its manufacturer, is not guaranteed or endorsed by the publisher.

## Footnotes

1
www.biocloud.net
