# Supplementary material for: Resveratrol Improves Growth Performance, Intestinal Morphology, and Microbiota Composition and Metabolism in Mice
Source: Front Microbiol. 2021 Sep 3;12:726878. doi: 10.3389/fmicb.2021.726878 (PMC8446547; doi:10.3389/fmicb.2021.726878)
Supplement: Supplementary file 1 [file Data_Sheet_1.docx]

**Supplementary Table 1** **Difference of bacterial communities in weaned C5 mice at the genus level**

| **genus** | **Mean (Con^1^)** | **Mean(RSV10^2^)** | **Mean(RSV20^3^)** | **Mean(RSV50^4^)** |
| --- | --- | --- | --- | --- |
| *Acinetobacter* | 0.00E+00 | 7.70E-05 | 0.00E+00 | 2.23E-03 |
| *Butyricicoccus* | 2.90E-06 | 1.59E-03 | 6.88E-04 | 1.83E-03 |
| *Lactobacillus* | 1.22E-03 | 3.01E-03 | 2.18E-02 | 5.96E-03 |
| *Roseburia* | 1.63E-03 | 1.79E-02 | 4.14E-03 | 1.53E-02 |
| *Ruminococcus_1* | 5.91E-06 | 6.80E-04 | 1.15E-03 | 3.73E-03 |
| *Streptococcus* | 2.90E-06 | 9.14E-05 | 6.13E-05 | 1.52E-04 |
| *Anaerotruncus* | 1.44E-05 | 9.72E-03 | 3.84E-03 | 7.04E-03 |
| *Bacteroides* | 2.90E-01 | 7.29E-03 | 1.20E-02 | 4.49E-03 |
| *Bifidobacterium* | 5.88E-06 | 2.05E-04 | 7.92E-04 | 1.20E-03 |

^1^Con = control group, ^2^RSV10 = 10 mg/kg RSV, ^3^RSV20 = 20 mg/kg RSV, and ^4^RSV50 = 50 mg/kg RSV. The p values were calculated using Metastats software. All the data are expressed as the means of six samples.

**Supplementary Figure 1**


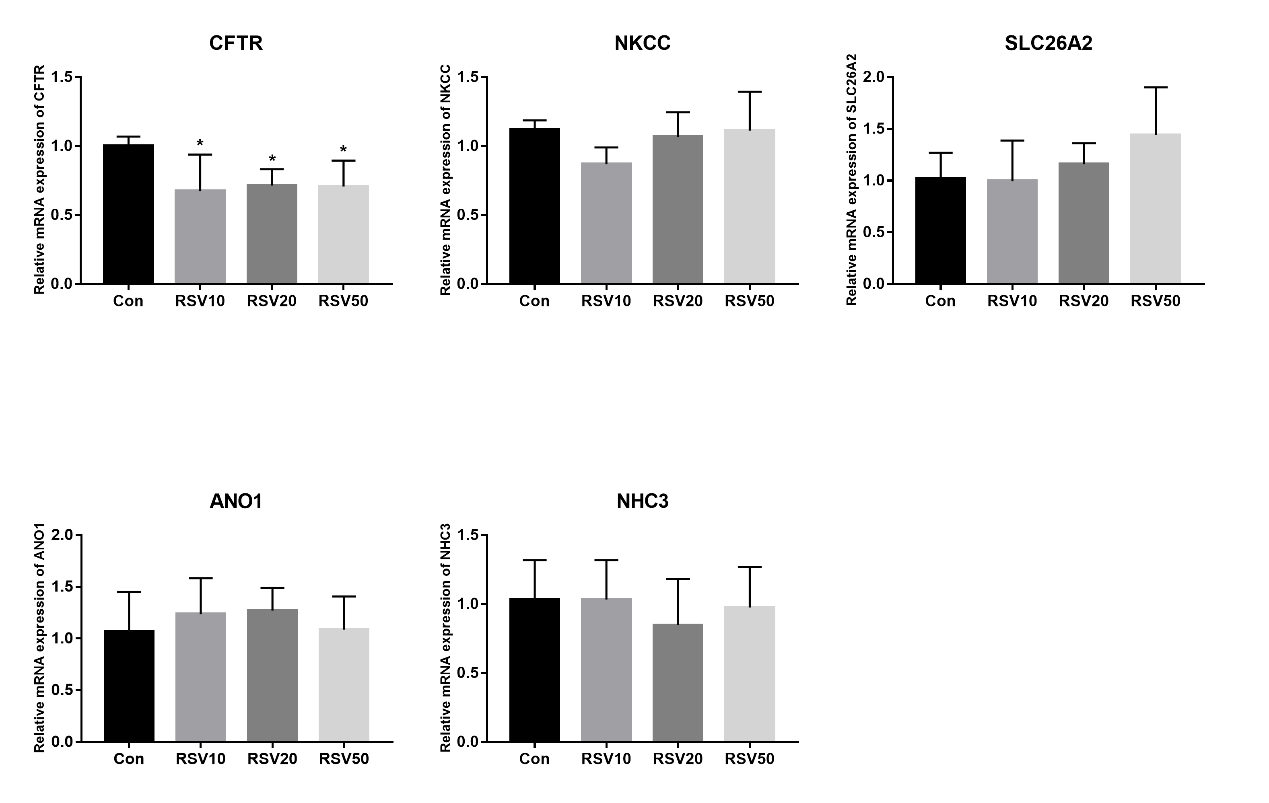


**Supplementary Figure 1. Effect of RSV on the expression of ion chanel-related genes in the ileum of weaned mice.** The relative mRNA expression of CFTR, NKCC, SLC26A2, ANO1, and NHC3 genes was detected by qRT-PCR. Con = control group, RSV10 = 10 mg/kg RSV, RSV20 = 20 mg/kg RSV, and RSV50 = 50 mg/kg RSV. *Means with single asterisks are significantly different (p < 0.05) from values of control group, and all the data are expressed as the means ± SEM.

**Supplementary Figure 2**


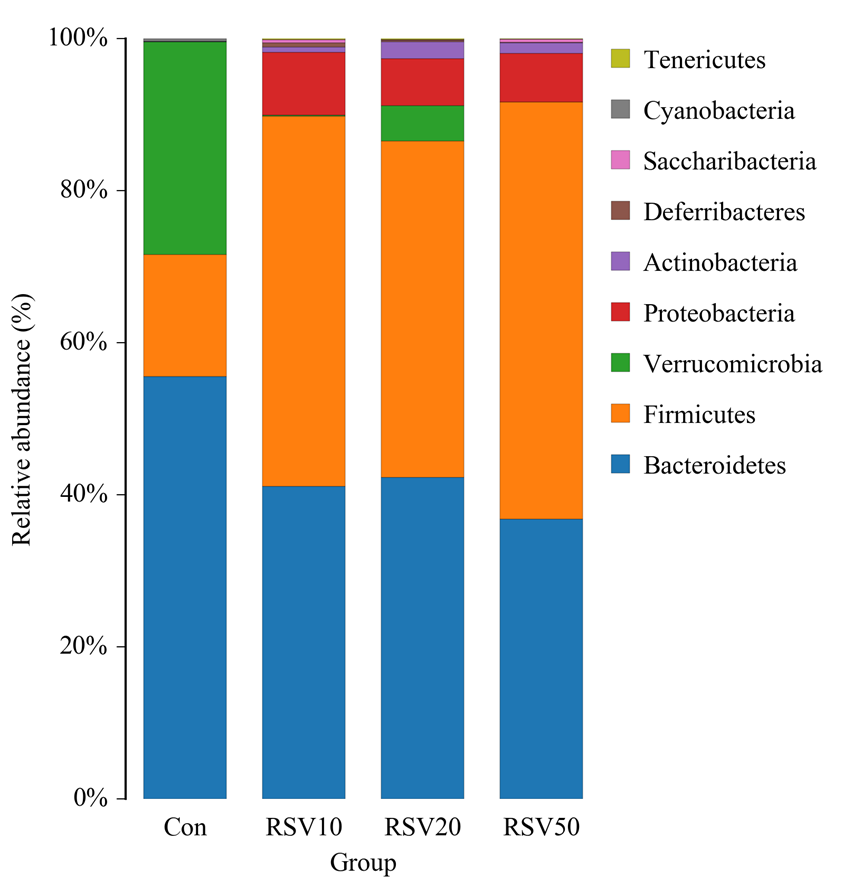


**Supplementary Figure 2. Effect of RSV on the bacterial communities in the cecum content of weaned mice at the phylum level**. Con = control group, RSV10 = 10 mg/kg RSV, RSV20 = 20 mg/kg RSV, and RSV50 = 50 mg/kg RSV.
